# Association of Estimated Pulse Wave Velocity with Chronic Kidney Disease Risk: A Machine Learning Analysis Based on NHANES and CHARLS

**DOI:** 10.3390/healthcare14183125

**Published:** 2026-09-21

**Authors:** Yunxiu Wu, Ye Yuan, Yaoyao Li, Ruizhao Li, Juan Pang

**Affiliations:** 1 Department of Endocrinology, Guangdong Provincial People’s Hospital, Zhuhai Hospital (Jinwan Central Hospital of Zhuhai), Zhuhai 519000, China; 15917904323@163.com; 2 Department of Nephrology, Guangdong Provincial People’s Hospital, Zhuhai Hospital (Jinwan Central Hospital of Zhuhai), Zhuhai 519000, China; 13631293759@139.com; 3 Hospital Office, Guangdong Provincial People’s Hospital, Zhuhai Hospital (Jinwan Central Hospital of Zhuhai), Zhuhai 519000, China; liyy19820318@163.com

**Keywords:** chronic kidney disease, estimated pulse wave velocity, NHANES, CHARLS, machine learning, SHAP

## Abstract

**Background:** Estimated pulse wave velocity (ePWV) is a non-invasive marker of arterial stiffness with potential relevance for chronic kidney disease (CKD) risk assessment. This study aimed to investigate the association between ePWV and CKD risk in the US and Chinese populations and to evaluate its discriminative performance using machine learning approaches. **Methods:** Data were obtained from the National Health and Nutrition Examination Survey (NHANES, 2005–2018, weighted *n* ≈ 100.9 million) and the China Health and Retirement Longitudinal Study (CHARLS, 2011–2015, *n* = 21,853). In NHANES, CKD was defined according to the 2021 KDIGO criteria as estimated glomerular filtration rate (eGFR) < 60 mL/min/1.73 m^2^ or a urinary albumin-to-creatinine ratio (ACR) ≥ 30 mg/g; in CHARLS, where urinary albumin was not measured, CKD was defined by the eGFR criterion alone, and this difference in case definition was taken into account when interpreting the results. Logistic regression and restricted cubic spline models were used to examine the association between ePWV and CKD, with subgroup analyses stratified by demographic and clinical characteristics. Multiple machine learning models were developed in NHANES and externally validated in CHARLS; model discrimination was assessed using the area under the receiver operating characteristic curve (AUROC), and feature importance was interpreted using SHapley Additive exPlanations (SHAP) values. **Results:** Higher ePWV was consistently associated with higher odds of CKD in both cohorts (NHANES: odds ratio [OR] = 1.505 per 1 m/s, 95% confidence interval [CI] = 1.459–1.552; CHARLS: OR = 1.434 per 1 m/s, 95% CI = 1.368–1.503; both *p* < 0.0001), with dose–response relationships observed. Formal interaction tests confirmed significant effect modification by sex (both cohorts) and by BMI and diabetes (NHANES only). Point estimates were higher in men and in NHANES obese and diabetic subgroups, but interactions across smoking and alcohol strata were not statistically significant. Among the machine learning models evaluated, discrimination was moderate and comparable across algorithms (LightGBM: AUROC = 0.804 in internal validation and 0.793 in external validation), and DeLong tests showed no significant difference between LightGBM and XGBoost (*p* = 0.0655 and 0.0684, respectively). SHAP analysis identified ePWV as the highest-ranking feature, surpassing uric acid, lipid levels, and diabetes history. Using the Youden index, the optimal ePWV cutoff for identifying CKD was 10.15 m/s in NHANES (sensitivity 0.658, specificity 0.695) and 10.568 m/s in CHARLS (sensitivity 0.658, specificity 0.718). **Conclusions:** Elevated ePWV is significantly associated with eGFR-defined CKD across the US and Chinese populations studied. These findings support ePWV as a potentially useful marker for CKD risk stratification; however, given the cross-sectional design of both cohorts, its predictive value requires confirmation in prospective studies. It is important to emphasize that no non-invasive calculated metric can replace direct measurement of serum creatinine and urinalysis for identifying individuals at risk of CKD in routine clinical practice.

## 1. Background

Chronic kidney disease (CKD) is a major global public health concern with increasing prevalence. It is a significant risk factor for cardiovascular disease, end-stage renal disease (ESRD), and all-cause mortality [1]. According to the Global Burden of Disease (GBD) 2021 report, more than 800 million individuals worldwide are affected by CKD, including approximately 130 million in China, representing a substantial disease burden [2]. However, early-stage CKD is often clinically silent, and diagnosis is commonly delayed until significant renal function loss has occurred, resulting in missed opportunities for timely intervention [2]. Therefore, there is an urgent need to identify non-invasive and effective markers for the early identification of individuals at increased CKD risk, which is of great importance to both clinical practice and public health.

Arterial stiffness is recognized as a key pathophysiological mechanism underlying chronic kidney disease (CKD) [3]. Pulse wave velocity (PWV), the gold standard for assessing arterial stiffness, has been consistently associated with both cardiovascular events and CKD in previous studies [4]. Elevated PWV reflects not only reduced vascular elasticity and increased hemodynamic load but also indicates a higher risk of microvascular injury within the kidneys [5]. However, conventional PWV measurement requires specialized equipment and trained personnel, making it costly and impractical for large-scale population screening. In recent years, an estimated form of PWV (ePWV), which is derived from readily available clinical parameters such as age and mean arterial pressure, has been proposed [6]. ePWV offers advantages of being non-invasive, simple to obtain, and highly reproducible, and has shown initial promise for cardiovascular risk stratification [7]. Nevertheless, its utility in risk assessment for CKD remains insufficiently investigated.

However, with the advancement of big data and artificial intelligence, machine learning approaches have demonstrated significant advantages in developing disease prediction models [8]. Compared with traditional statistical methods, machine learning is capable of capturing complex nonlinear relationships and uncovering interactions among multidimensional features, thereby enhancing predictive performance. A range of ensemble learning algorithms—such as Random Forest (RF), Extreme Gradient Boosting (XGBoost), and Light Gradient Boosting Machine (LightGBM)—have been widely applied in risk prediction for cardiovascular, oncological, and metabolic diseases [9,10,11]. In parallel, the development of interpretable machine learning has provided tools to improve model transparency. Among them, SHapley Additive exPlanations (SHAP) enables quantification of each feature’s contribution to the model output [12], revealing not only its direction of effect but also its pattern of influence, thus offering clinically interpretable insights for decision-making.

To date, studies investigating the association between estimated pulse wave velocity (ePWV) and CKD risk remain limited, often confined to single populations and lacking external validation across diverse cohorts. Moreover, there is a paucity of research employing machine learning approaches to systematically evaluate the utility of ePWV and its relative importance compared with traditional risk factors in CKD risk stratification. In this context, to our knowledge, the present study is among the first to integrate data from two large, nationally representative cohorts—the US National Health and Nutrition Examination Survey (NHANES) and the China Health and Retirement Longitudinal Study (CHARLS)—to comprehensively examine the relationship between ePWV and CKD risk. Multiple machine learning algorithms were applied to develop and compare classification models, with external validation performed in an independent cohort. Furthermore, the SHapley Additive exPlanations (SHAP) framework was used to interpret feature contributions and interaction patterns, aiming to provide novel insights for CKD risk stratification and early detection.

## 2. Materials and Methods

### 2.1. Study Design Overview

This study systematically evaluated the association between estimated pulse wave velocity (ePWV) and chronic kidney disease (CKD) using two nationally representative observational cohorts: the National Health and Nutrition Examination Survey (NHANES) in the United States and the China Health and Retirement Longitudinal Study (CHARLS). To ensure data quality and comparability across cohorts, we applied uniform inclusion and exclusion criteria [13,14]. Participants were eligible if they were aged ≥45 years and had complete information on blood pressure and serum creatinine, as well as urinary albumin for the NHANES cohort. Individuals were excluded if they lacked key variables or presented with extreme ePWV values. CKD was defined according to the core criteria of the Kidney Disease: Improving Global Outcomes (KDIGO) 2021 guidelines [15], which require either a reduced glomerular filtration rate or albuminuria. eGFR was calculated using the CKD-EPI equation. In NHANES, which measured both serum creatinine and urinary albumin, CKD was defined as eGFR < 60 mL/min/1.73 m^2^ or a urinary albumin-to-creatinine ratio (ACR) ≥ 30 mg/g; in CHARLS, where urinary albumin was not available, CKD was defined as eGFR < 60 mL/min/1.73 m^2^ alone. All remaining participants were considered non-CKD. Application of these criteria to the baseline datasets of the two cohorts yielded the final analytical samples. All statistical analyses were performed using R software version 4.4.3 and Python version 3.11.5.

### 2.2. Two Extensive Observational Cohort Studies

The National Health and Nutrition Examination Survey (NHANES) is a nationally representative survey of adults and children in the United States, designed to assess health and nutritional status through demographic information, dietary habits, laboratory tests, physical examinations, and health-related questionnaires. This study utilized pooled data from the continuous NHANES cycles covering 2005–2018. The total number of participants who completed the Mobile Examination Center (MEC) interview and examination across these combined cycles was 70,190 before the application of study-specific exclusion criteria. According to the predefined criteria [13], we sequentially excluded individuals aged <45 years (*n* = 47,268), those without blood pressure measurements required for ePWV calculation (*n* = 4002), those lacking serum creatinine or urinary albumin-to-creatinine ratio data (*n* = 1011), and those with extreme ePWV values (*n* = 10). The final analytic sample consisted of 17,890 participants. The NHANES protocol was approved by the Ethics Review Board of the National Center for Health Statistics, and all participants provided written informed consent before data collection. All statistical analyses incorporated the sampling weights, strata, and primary sampling unit variables provided by NHANES to ensure nationally representative estimates for the non-institutionalized middle-aged and older U.S. population.

The China Health and Retirement Longitudinal Study (CHARLS) is a nationally representative longitudinal survey aimed at evaluating the health and socioeconomic status of middle-aged and older adults in China. We utilized data from the 2011 and 2015 survey waves, with an initial sample size of 57,405 participants. 

For this cross-sectional analysis, data from the two waves were pooled; for participants who completed both surveys, only the 2011 baseline record was retained to avoid duplicate measurements and within-participant correlations.

Following the same criteria [14], we excluded individuals younger than 45 years (*n* = 2180), those without sex information (*n* = 19), those missing blood pressure measurements for ePWV calculation (*n* = 32,927), those lacking serum creatinine measurements required for eGFR calculation (*n* = 243), and those with extreme ePWV values (*n* = 183). The final analytical sample included 21,853 participants. The CHARLS protocol was approved by the Institutional Review Board of Peking University, and written informed consent was obtained from all the participants.

### 2.3. Definition of Chronic Kidney Disease (CKD)

CKD was defined as a binary outcome (yes/no) according to the 2021 Kidney Disease: Improving Global Outcomes (KDIGO) guidelines, which require either a reduced glomerular filtration rate or evidence of albuminuria. eGFR was calculated using the CKD-EPI equation. In NHANES, where both serum creatinine and urinary albumin were measured, CKD was defined as eGFR < 60 mL/min/1.73 m^2^ or a urinary albumin-to-creatinine ratio (ACR) ≥ 30 mg/g, consistent with the full KDIGO definition; participants meeting neither criterion were classified as non-CKD. In CHARLS, urinary albumin was not measured, and CKD could therefore be ascertained only by the eGFR criterion (eGFR < 60 mL/min/1.73 m^2^ alone) [15]. This inter-cohort difference in case definition was explicitly acknowledged: CHARLS does not capture individuals who have albuminuria with preserved eGFR, and therefore CKD prevalence, all descriptive statistics, and all model-derived estimates differ between the two cohorts by definition and are not directly comparable in absolute terms. Every result reported in this manuscript was generated using the definition stated above for the respective cohort, and all analyses within each cohort are internally consistent with its own case definition.

### 2.4. Estimation of Pulse Wave Velocity (ePWV)

In this study, estimated pulse wave velocity (ePWV) was calculated using an established equation derived from reference values for arterial stiffness. The calculation was based on age and mean blood pressure (MBP), using the following formula:

ePWV = 9.587 − (0.402 × Age) + (4.560 × 10^−3^ × Age^2^) − (2.621 × 10^−5^ × Age^2^ × MBP) + (3.176 × 10^−3^ × Age × MBP) − (1.832 × 10^−2^ × MBP)

MBP was calculated from the systolic and diastolic blood pressures using the following formula: [13].

MBP = Diastolic Blood Pressure (DBP) + 0.4 × [Systolic Blood Pressure (SBP) − DBP]

### 2.5. Covariates

Covariates were pre-specified based on prior studies and biological plausibility [13] and included demographic characteristics (age, sex, race/ethnicity, marital status, educational attainment, and poverty income ratio (PIR) or income category), anthropometric and biochemical measurements (body mass index (BMI), uric acid (UA), total cholesterol (TC), triglycerides (TG), low-density lipoprotein cholesterol (LDL), alanine aminotransferase (ALT), aspartate aminotransferase (AST), hemoglobin (HGB), and estimated glomerular filtration rate (eGFR), which was used only for outcome definition and sensitivity analysis), lifestyle factors (smoking status, alcohol consumption, and physical activity), and self-reported history of chronic conditions including hypertension, diabetes, and dyslipidemia. SBP, DBP, and MBP were not included as covariates in the multivariable models because they are already incorporated into the ePWV calculation; including them would introduce collinearity.

### 2.6. Weighting and Representativeness

The NHANES employs a multistage, stratified, cluster sampling design to ensure national representativeness. In this study, all analyses incorporated the complex survey design parameters provided by the NHANES, including sampling weights, strata, and primary sampling units. Specifically, the 2-year mobile examination center (MEC) weight (WTMEC2YR) was used; when combining seven survey cycles from 2005 to 2018, the weight was adjusted as WTMEC2YR × 1/7 in accordance with the NHANES analytic guidelines. The SDMVSTRA (stratum variable) and SDMVPSU (primary sampling unit) were also included to produce nationally representative estimates of means, standard errors, and confidence intervals [16].

CHARLS uses a multistage stratified sampling design to capture a nationally representative sample of middle-aged and older adults in China. In the present study, analyses were conducted without weighting, consistent with prior CHARLS publications [17]; therefore, the results reflect the general characteristics of the Chinese middle-aged and older population.

### 2.7. Statistical Analysis

All statistical analyses were conducted using R software version 4.4.3. For the NHANES cohort, appropriate sample weights were applied to account for the complex, multistage stratified sampling design, whereas analyses for the CHARLS cohort were performed without weighting. Continuous variables with a normal distribution were presented as mean ± standard deviation (SD) and compared using independent sample *t*-tests. Non-normally distributed variables were expressed as medians with interquartile ranges (IQR) and compared using the Wilcoxon rank-sum test. Categorical variables were presented as counts and percentages, and intergroup comparisons were performed using the chi-square test.

To evaluate the association between ePWV and CKD, multivariable logistic regression models were applied. Three models were progressively constructed: Model 1 was unadjusted; Model 2 was adjusted for sex, residence, marital status, and educational level; and Model 3 was further adjusted for UA, TG, HGB, TC, BMI, LDL-C, hypertension, dyslipidemia, diabetes, alcohol consumption, smoking status, and metabolic equivalents (MET). SBP, DBP, and MBP were not included as covariates because they were already incorporated into the ePWV calculation. Restricted cubic spline (RCS) functions were employed to explore potential nonlinear dose–response relationships between ePWV and CKD risk. Four knots were placed at the 5th, 35th, 65th, and 95th percentiles of the ePWV distribution (NHANES: 7.017, 8.783, 10.461, 13.265; CHARLS: 6.913, 8.683, 10.216, 13.095), with the median ePWV as the reference (NHANES: 9.564; CHARLS: 9.411). Analyses were repeated across the three models (Model 1–3) to assess the robustness of the associations.

Participants were stratified by sex (male vs. female), body mass index (normal, overweight, obese), alcohol consumption status (no vs. yes), smoking status (no vs. yes), hypertension (no vs. yes), dyslipidemia (no vs. yes), and diabetes (no vs. yes). Within each subgroup, three multivariable logistic regression models (Model 1–3) were constructed to evaluate the association between ePWV and CKD. Odds ratios (ORs) and corresponding 95% confidence intervals (CIs) were reported. To assess potential effect modification, interaction terms were added to the multivariable models, and *p* values for interaction were calculated accordingly [18].

### 2.8. Machine Learning Analysis

In the machine learning analysis, ePWV and related covariates were used as input features, with CKD status as the outcome variable. The following variables were explicitly excluded from the feature set to prevent target leakage: eGFR and serum creatinine, as both are directly used to define the outcome. Multiple machine learning algorithms were implemented, including Random Forest, XGBoost, LightGBM, AdaBoost, Naive Bayes, and Decision Tree models [19].

The data from the NHANES cohort were randomly split into training and validation sets in a 7:3 ratio, and external validation was performed using the CHARLS cohort. Continuous variables were standardized using the mean and standard deviation from the training set only; categorical variables were encoded using one-hot encoding. Missing values were imputed using the training set median for continuous variables and the training set mode for categorical variables.

Hyperparameter tuning was performed via a 5-fold grid search within the training set only. Class imbalance was addressed using balanced class weights in all tree-based models. No validation data were used for model training or tuning.

Model performance was evaluated based on accuracy, area under the receiver operating characteristic curve (AUROC) with 95% confidence intervals, F1-score, Matthews correlation coefficient (MCC), sensitivity, and specificity, with average values used to represent overall performance. Discrimination ability was assessed using ROC and precision-recall (PR) curves; calibration was evaluated using calibration curves and Brier scores. Clinical utility was further assessed via decision curve analysis (DCA). Residual Q–Q plots were included as an ancillary tool to visually assess the distributional assumptions of the model residuals, complementing the primary evaluation metrics. Five-fold cross-validation was applied to enhance the robustness of the results, and generalizability was assessed in the external validation set [20]. To explore the contribution of each feature to CKD classification, SHAP (SHapley Additive exPlanations) values were calculated and visualized to quantify and interpret the importance and impact of each variable on model predictions.

## 3. Result

### 3.1. Baseline Characteristics of the Study Population

This study included participants from two nationally representative cohorts: the United States NHANES (Table 1) and the Chinese CHARLS (Table 2). In the NHANES population, a weighted total of 100,907,775 individuals were analyzed, of whom 21,604,517 were identified as having CKD, yielding a prevalence of 21.4%. In the CHARLS cohort, 21,853 participants were included, among whom 746 had CKD, corresponding to a prevalence of 3.4%. Across both populations, individuals with CKD were significantly older than those without CKD (*p* < 0.001). ePWV levels were markedly elevated in the CKD group, and CKD prevalence increased significantly across ascending ePWV quartiles (both *p* for trend <0.001). In the highest quartile of ePWV (Q4), the prevalence of CKD reached 51.58% in NHANES and 8.22% in CHARLS. Among all CHARLS participants with CKD, 60.19% were in Q4. Regarding kidney function, eGFR levels were significantly lower in CKD individuals compared to their non-CKD counterparts. In terms of comorbidities, the NHANES CKD group exhibited significantly higher prevalence rates of hypertension (67.63%), diabetes (30.19%), and dyslipidemia (55.57%) than the non-CKD group (all *p* < 0.001). Similarly, in CHARLS, participants with CKD had higher rates of hypertension (50.00%), diabetes (15.15%), and dyslipidemia (19.57%) (all *p* < 0.001). Sociodemographic characteristics revealed that within the CHARLS cohort, the CKD group had a higher proportion of individuals with low educational attainment, especially among those with CKD (35.12% vs. 27.00%, *p* < 0.001). Urban residency was also significantly more common in the CKD group (*p* < 0.001). Although the overall education and income levels were higher in NHANES, a significantly greater proportion of CKD participants fell into the low-income category (*p* < 0.001). Taken together, individuals with CKD in both populations were characterized by older age, elevated ePWV, impaired kidney function, and clustering of cardiometabolic risk factors.

### 3.2. Association Between ePWV and CKD Risk

In both the U.S. NHANES and Chinese CHARLS populations, logistic regression analyses demonstrated a significant association between elevated ePWV and increased CKD risk (Table 3 and Table 4). In NHANES, each 1 m/s increase in ePWV was associated with an OR of 1.505 (95% CI: 1.459–1.552); in CHARLS, the corresponding OR was 1.434 (95% CI: 1.368–1.503) (both *p* < 0.0001). Stratified analyses further revealed a stepwise increase in CKD risk with rising ePWV levels in both populations, with trend tests consistently reaching statistical significance (*p* < 0.0001). Participants in the highest quartile of ePWV (Q4) had markedly higher CKD risk than those in the lowest quartile (Q1). Taken together, despite differences in demographic and clinical characteristics between NHANES and CHARLS, both cohorts indicated that elevated ePWV was associated with CKD, and exhibited a dose–response relationship in NHANES, while in CHARLS the association was largely age-driven.

### 3.3. Dose–Response Relationship Between ePWV and CKD Risk

**Figure 1:**
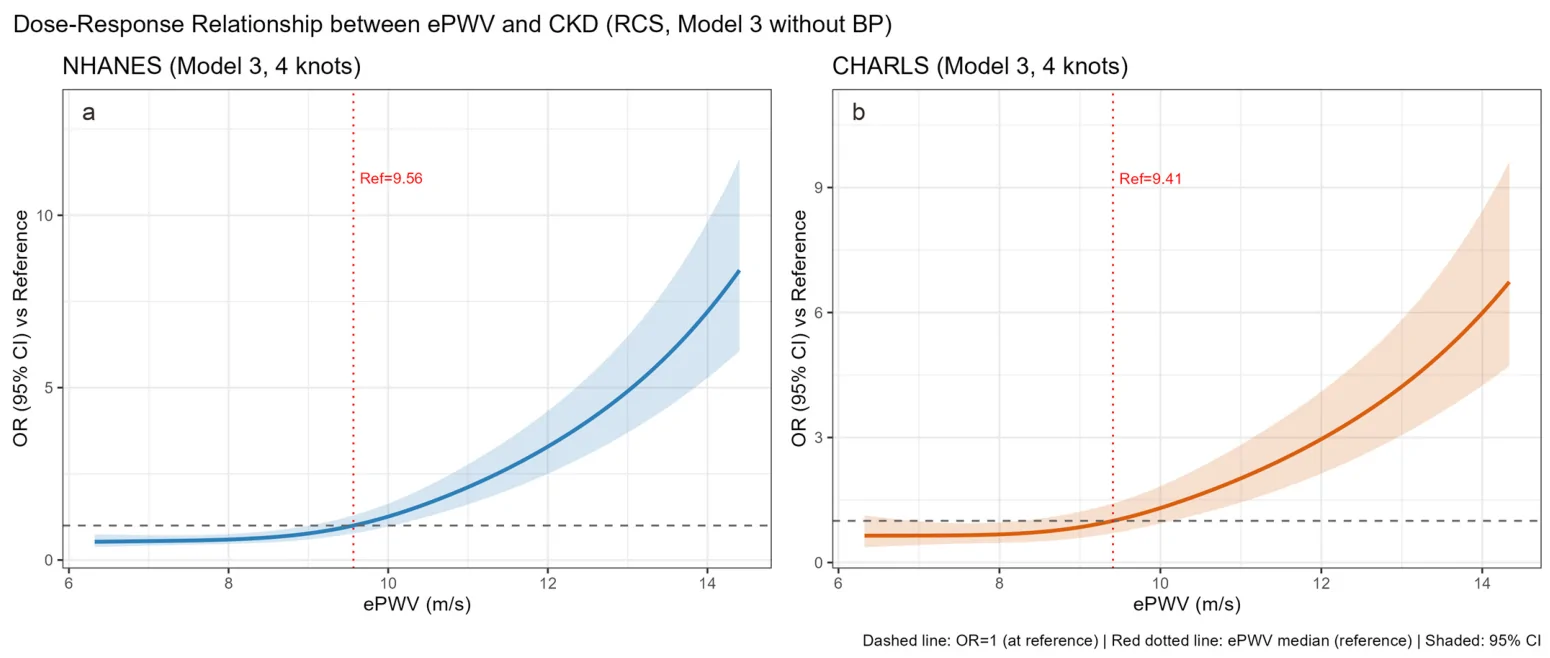
Dose–response relationship between ePWV and CKD risk based on restricted cubic spline models. Panel (**a**): NHANES; Panel (**b**): CHARLS. The models were adjusted for covariates in Model 3. The reference ePWV was set at the median (9.564 m/s for NHANES, 9.411 m/s for CHARLS). Shaded areas represent 95% confidence intervals. *p* for nonlinearity was <0.001 for NHANES and 0.071 for CHARLS.

The dose–response relationship between ePWV and CKD risk was assessed using restricted cubic spline (RCS) models(Table 5). In the NHANES cohort, elevated ePWV was significantly and positively associated with CKD, exhibiting a distinct nonlinear pattern (*p* for nonlinearity < 0.001; *p* for overall association < 0.001; Figure 1a). In the CHARLS cohort, the association appeared more gradual and did not show significant nonlinearity (*p* for nonlinearity = 0.071), although the overall association remained statistically significant (*p* for overall < 0.001; Figure 1b). Overall, both analyses support the role of ePWV as a predictive marker for CKD risk.

After additional adjustment for age, the association was markedly attenuated in the CHARLS cohort (Table 6) (OR = 0.997, 95% CI: 0.921–1.080, *p* = 0.947), indicating that ePWV is not a fully independent risk factor once age is accounted for.

### 3.4. Stability and Heterogeneity of the ePWV–CKD Association Across Subgroups

**Figure 2:**
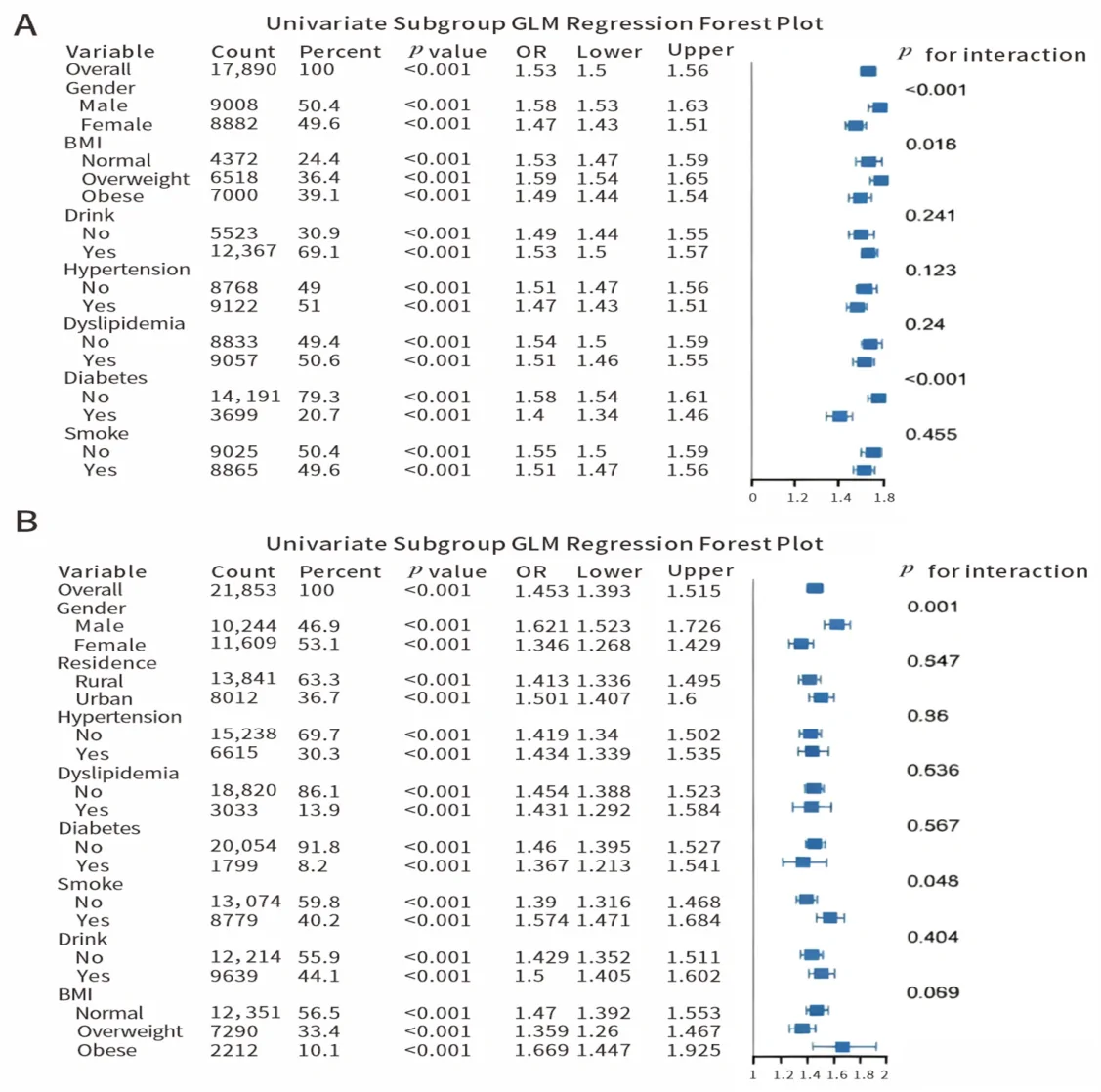
Subgroup analyses of the association between ePWV and CKD. Panel (**A**): NHANES; Panel (**B**): CHARLS. ORs were obtained from Model 3 (fully adjusted). Squares represent point estimates; horizontal lines represent 95% confidence intervals. The *P* interaction values are presented in Table 4.

Stratified analyses based on both the NHANES (Figure 2A) and CHARLS (Figure 2B) cohorts consistently demonstrated that elevated ePWV was associated with a higher prevalence of CKD. Specifically, each 1 m/s increase in ePWV was associated with an OR of 1.579 (95% CI: 1.507–1.655) in NHANES males and 1.441 (95% CI: 1.381–1.504) in NHANES females; in CHARLS, OR = 1.553 (95% CI: 1.453–1.659) in males and 1.318 (95% CI: 1.233–1.409) in females.

Formal interaction tests confirmed significant effect modification by sex in both cohorts (NHANES *p*_interaction = 0.012; CHARLS *p*_interaction < 0.001) and by BMI in NHANES (*p*_interaction = 0.025). No significant interactions were observed for smoking, drinking, hypertension, or dyslipidemia (all *p*_interaction > 0.05), except for diabetes in NHANES (*p*_interaction = 0.006). Additional subgroup analyses in NHANES showed a more pronounced association among non-diabetic individuals (OR = 1.52 vs. 1.45, *p* for interaction = 0.006), while CHARLS highlighted particularly strong associations among smokers (OR = 1.52) and individuals with obesity (OR = 1.63). Importantly, the presence of hypertension or dyslipidemia did not significantly alter the strength of the ePWV-CKD association in either cohort. These findings suggest that ePWV is associated with CKD across subgroups; formal interaction tests confirmed statistically significant effect modification by sex (both cohorts) and by BMI and diabetes in NHANES, whereas differences across smoking and alcohol strata were not significant for interaction and should be interpreted with caution.

To address the concern that ePWV may not provide additional information beyond its components (age and blood pressure), we compared the discriminative performance of models containing age only, blood pressure only, ePWV only, and all three combined (Table 7). In NHANES, adding ePWV to age improved the AUROC from 0.726 (95% CI: 0.717–0.734) to 0.735 (95% CI: 0.727–0.744), with significant improvements in NRI and IDI (Table 8) (NRI = 0.186, *p* < 0.001; IDI = 0.015, *p* < 0.001). In CHARLS, adding ePWV to age improved the AUROC from 0.759 (95% CI: 0.741–0.777) to 0.761 (95% CI: 0.743–0.779); while the NRI was not statistically significant (0.009, *p* = 0.800), the IDI showed significant improvement (0.010, *p* < 0.001). Using the Youden index, the optimal ePWV cutoff for discriminating CKD was 10.15 m/s in NHANES and 10.568 m/s in CHARLS (Table 9; Supplementary Figure S1).

### 3.5. Comparative Model Performance of Machine Learning Algorithms for CKD Classification

**Figure 3:**
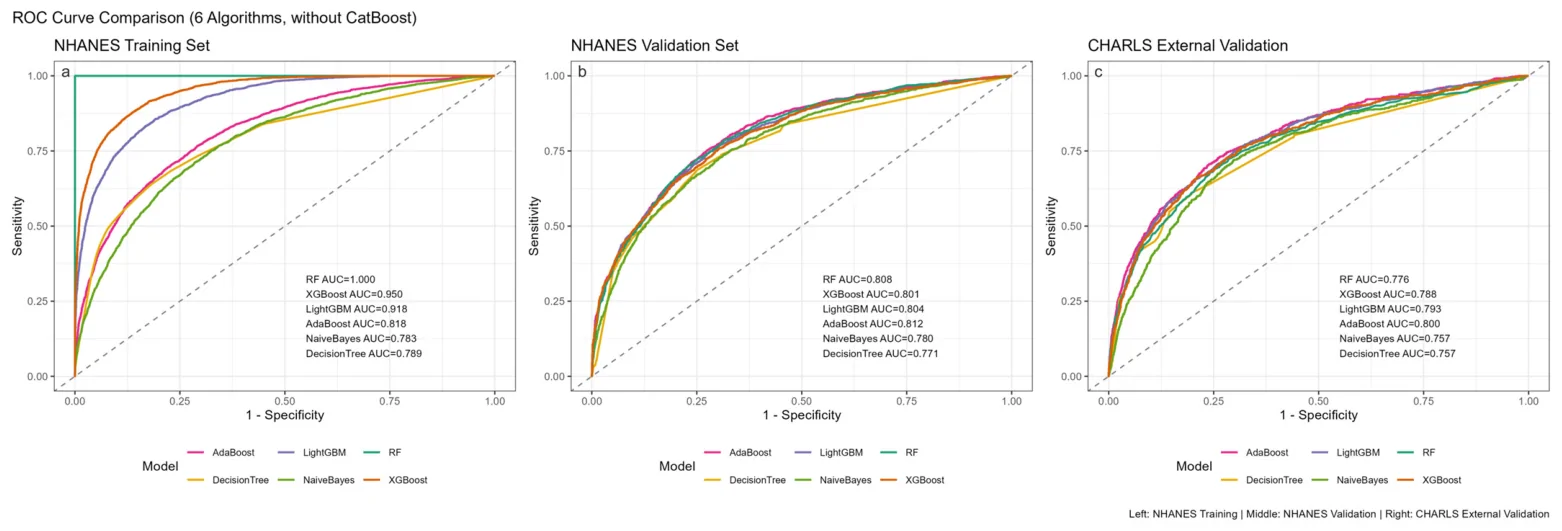
ROC curves of machine learning models for CKD classification. Panel (**a**): NHANES training set; Panel (**b**): NHANES validation set; Panel (**c**): CHARLS external validation set.

Using data from the NHANES cohort (randomly split into training and validation sets in a 7:3 ratio) and the CHARLS cohort (as an external validation set), we compared the performance of several machine learning algorithms for CKD classification, including Random Forest (RF), XGBoost, Naïve Bayes, Light Gradient Boosting Machine (LightGBM), decision tree, and AdaBoost (Table 10 and Table 11, Figure 3).

In the NHANES training set, LightGBM, XGBoost, and AdaBoost achieved accuracy rates exceeding 97%, with AUROCs approaching 1.0 and F1-scores above 0.98 (Table 10; Figure 3a). Naïve Bayes and Decision Tree models had accuracy rates below 80% (Table 10). In the NHANES validation set (Table 10; Figure 3b), LightGBM and XGBoost maintained high AUROCs of 0.804 (95% CI: 0.791–0.818) and 0.801 (95% CI: 0.787–0.815), respectively, with accuracy rates close to 75–79%. AdaBoost achieved an AUROC of 0.812 (95% CI: 0.798–0.825).

In the external validation using CHARLS data (Table 11; Figure 3c), LightGBM, XGBoost, and AdaBoost demonstrated consistent and robust performance, with AUROCs of 0.793 (95% CI: 0.775–0.809), 0.788 (95% CI: 0.771–0.805), and 0.800 (95% CI: 0.784–0.817), respectively.

Precision-recall (PR) curve analyses (Supplementary Figure S2) confirmed the consistent performance of LightGBM, XGBoost, and AdaBoost across all datasets. Calibration curves (Supplementary Figure S3) showed that LightGBM, XGBoost, and AdaBoost closely approximated the ideal diagonal line, whereas Naïve Bayes showed larger deviations. Decision curve analysis (Supplementary Figure S4) indicated greater net clinical benefit for LightGBM, XGBoost, and AdaBoost across a broad range of threshold probabilities. Finally, the residual Q–Q plots were included as a supplementary diagnostic tool to assess residual distribution patterns rather than as a primary performance metric; results showed that LightGBM, XGBoost, and AdaBoost had residual distributions closely aligned with theoretical expectations, while Naïve Bayes and Random Forest showed notable deviations (Supplementary Figure S5).

To assess the robustness of the LightGBM (LGBM) model, we performed five-fold cross-validation(Table 12).The results demonstrated that the model maintained consistent performance across all test folds with a mean AUROC of 0.7985 (95% CI: 0.7911–0.8045) across the five folds, the AUC values ranged from 0.784 to 0.806 (Fold 1–5: 0.7962, 0.8043, 0.8062, 0.8017, 0.7844). In the precision–recall (PR) curves, all folds achieved AUCs greater than 0.97. The calibration curves indicated good agreement between predicted and observed risks, with Brier scores ranging from 0.069 to 0.077. Decision curve analysis further confirmed that the model provided substantial net clinical benefit across a range of threshold probabilities. Additionally, residual Q–Q plots showed that the distribution of prediction residuals closely matched the theoretical distribution in each fold. These results collectively suggest that the LGBM model demonstrates strong robustness and calibration, with no evidence of systematic bias.

Taken together, these results indicate that the LightGBM model performed consistently across the internal validation, external validation, and cross-validation datasets, with moderate discrimination (AUROC approximately 0.79–0.80). DeLong tests showed no statistically significant difference between LightGBM and XGBoost in either the NHANES validation set (*p* = 0.0655) or the CHARLS external validation set (*p* = 0.0684)(Table 13); the model should therefore not be described as superior to the other algorithms.

### 3.6. Feature Importance and SHAP Visualization

**Figure 4:**
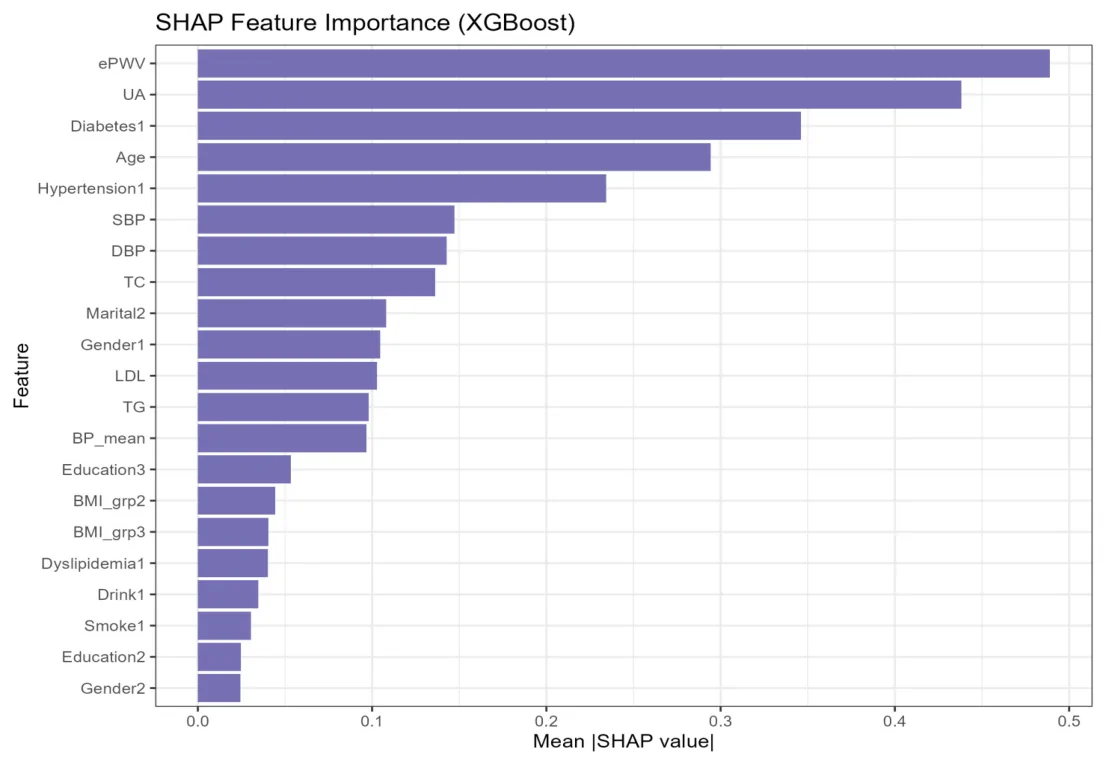
SHAP feature importance plot of the CKD classification model. The bars represent the mean absolute SHAP values. The ePWV showed the highest model-derived feature importance.

**Figure 5:**
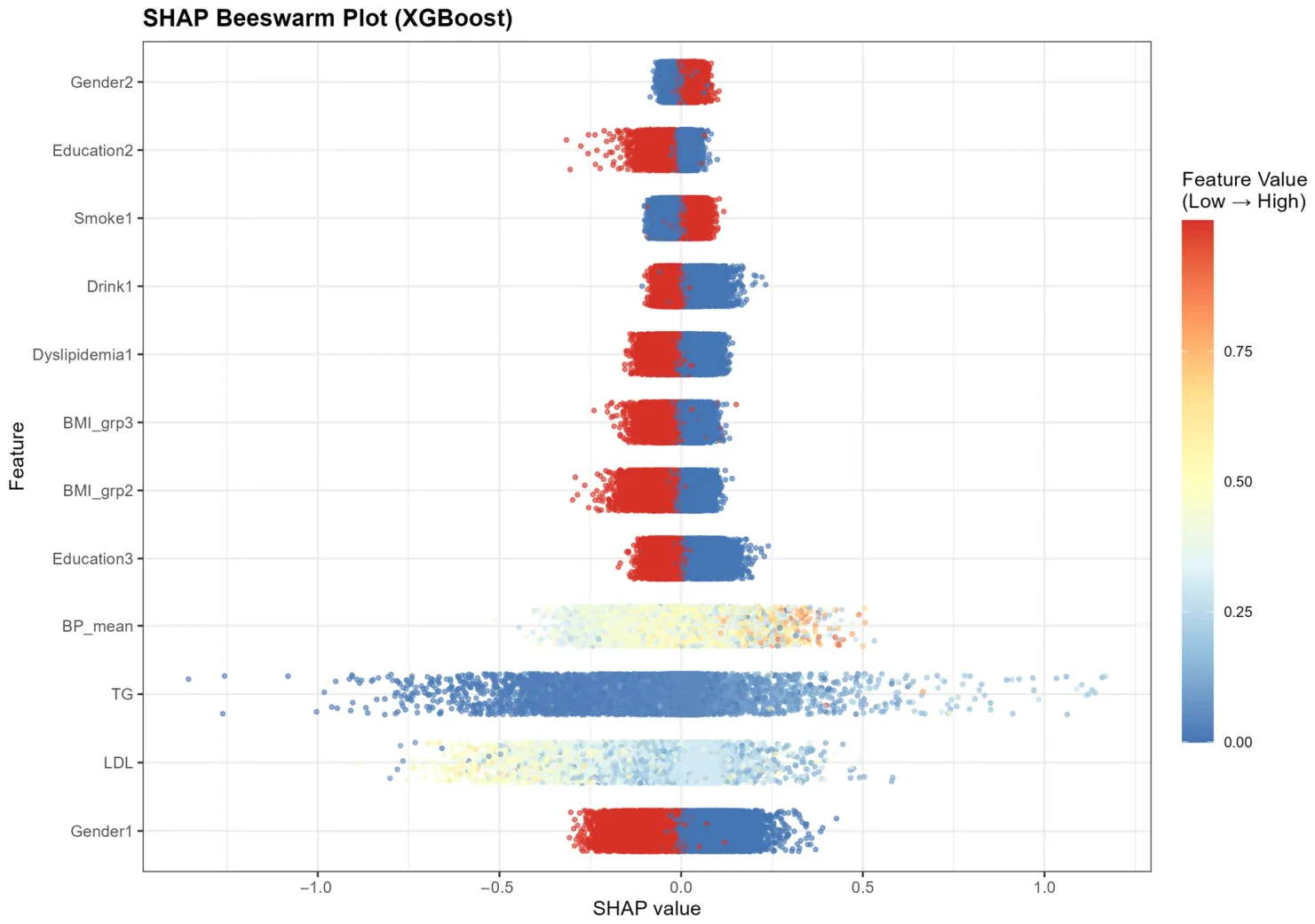
SHAP beeswarm plot of the top 12 features for the CKD classification model. Each dot represents one sample; the color represents the feature value (red: high; blue: low). The x-axis represents the SHAP value (impact on model output). Features ranked below the top 12 are not shown due to low contribution.

**Figure 6:**
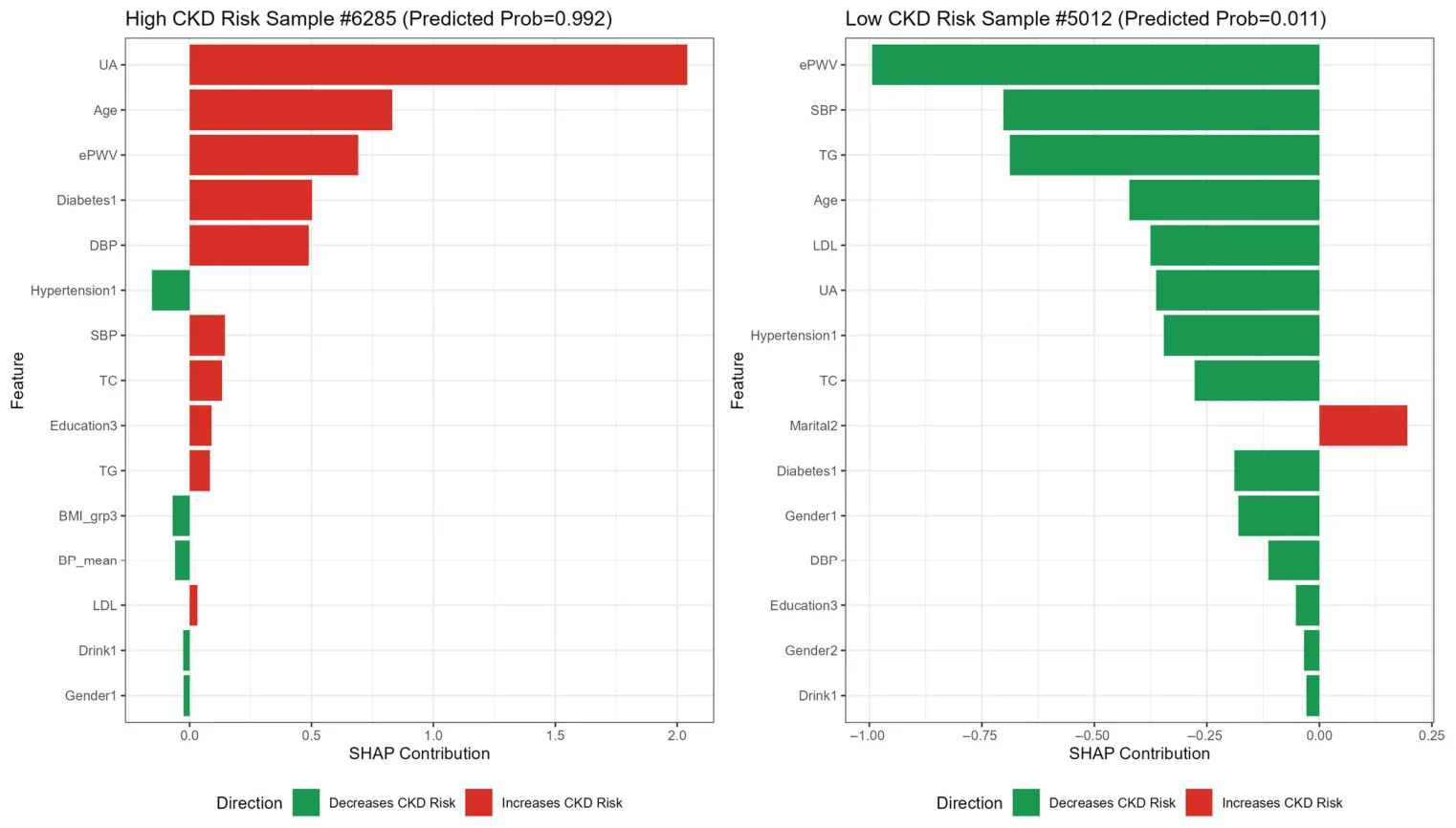
SHAP force plot for a representative individual. Features in red increase predicted CKD risk, whereas features in green decrease predicted risk.

In this study, a machine learning-based model was developed to predict CKD risk, and SHAP (SHapley Additive exPlanations) was applied for model interpretation. The global feature importance analysis revealed that ePWV ranked highest in model-derived feature importance, with a mean absolute SHAP value of 0.489 (16.1% of total importance), surpassing uric acid (0.438, 14.5%), diabetes (0.346, 11.4%), age (0.294, 9.7%), and hypertension (0.234, 7.7%) (Figure 4; Table 14). In terms of feature effect patterns, ePWV exhibited a clear positive dose–response relationship, where higher values significantly increased CKD risk and lower values were associated with reduced risk (Figure 5). At the individual prediction level, SHAP force plots demonstrated that the model’s risk assessment was driven by complex interactions among multiple features. While elevated ePWV increased CKD risk, protective factors such as the absence of diabetes and hypertension exerted negative effects that could partially offset the adverse impact (Figure 6).

## 4. Discussion

This study systematically investigated the association between estimated pulse wave velocity (ePWV) and chronic kidney disease (CKD) using two nationally representative and independent population-based cohorts, NHANES in the United States and CHARLS in China. The key findings can be summarized as follows: First, in multivariable-adjusted models, elevated ePWV was significantly associated with an increased risk of CKD, showing a clear dose–response relationship. This association was consistently observed across both Western and Eastern populations. Second, subgroup analyses revealed that the association between ePWV and CKD was stronger among men, individuals with obesity, and smokers. Finally, machine learning modeling combined with SHAP-based interpretability analysis demonstrated that ePWV was the highest-ranking feature in model-derived importance. The LightGBM model built upon this feature exhibited consistent and robust discriminative performance.

Extensive prior research has established arterial stiffness as an independent predictor of both the onset and progression of chronic kidney disease (CKD) [21,22,23]. As a simple and accessible surrogate marker, estimated pulse wave velocity (ePWV) has demonstrated associations across various populations and clinical settings in recent years. Analyses of NHANES data have revealed nonlinear or J-shaped associations between ePWV and CKD risk, as well as strong correlations between elevated ePWV and increased all-cause and cardiovascular mortality [24,25,26]. A study based on the MIMIC-IV database also indicated that elevated ePWV significantly predicted poor outcomes—such as increased in-hospital and one-year mortality—in critically ill patients with concurrent CKD and atherosclerosis [27].

Moreover, several studies suggest that the predictive ability of ePWV may be modified by individual characteristics such as sex, diabetes status, CKD stage, and height, all of which should be taken into account in clinical applications [28]. Mechanistically, ePWV has been shown to interact with Klotho protein levels, jointly contributing to CKD pathogenesis [29]. A strong correlation has also been reported between ePWV and carotid atherosclerotic plaque formation, particularly in patients with diabetes [30].

Beyond these vascular and renal mechanisms, the association between arterial stiffness and CKD may also be interpreted within a broader immunometabolic framework, in which metabolic stress, chronic low-grade inflammation, oxidative imbalance, mitochondrial dysfunction, and vascular injury interact through interconnected feedback mechanisms [31,32,33]. Such network-level interactions may contribute to endothelial and microvascular dysfunction and provide a biological context linking increased arterial stiffness to renal injury. This perspective is consistent with the emerging view that vascular, metabolic, inflammatory, and organ-specific disturbances should be considered as interconnected components of systemic pathophysiology.

Longitudinal cohort studies, such as ARIC, further support these findings by linking higher PWV levels to increased CKD incidence and faster declines in renal function over time [34].

To our knowledge, the present study is among the first to systematically evaluate the dose–response relationship and model robustness of ePWV for CKD across both U.S. and Chinese population-based datasets. Our results demonstrate that ePWV is consistently associated with CKD in diverse populations, exhibiting greater model-derived feature importance than many traditional risk factors in machine learning models. These findings provide external validation for ePWV and underscore its broad applicability and potential clinical value across different ethnic and health-status groups.

This study found that the association between ePWV and CKD risk was more pronounced in males, individuals with obesity, and smokers. One possible explanation lies in sex-specific vascular aging mechanisms. As highlighted by DuPont et al. in a systematic review, the progression of arterial stiffness differs by sex in relation to age, obesity, and hypertension, with sex hormones and structural vascular differences contributing to variations in arterial elasticity [35]. In addition, smoking is known to accelerate arterial stiffening by promoting inflammation and endothelial dysfunction, while comorbidities such as hypertension and diabetes further impair vascular health [36].

Notably, there is currently no strong evidence suggesting that traditional metabolic disorders such as diabetes or hypertension significantly modify the relationship between ePWV and CKD. This suggests that the association between ePWV and CKD is independent of conventional risk factors. From a clinical perspective, early identification of high-risk subgroups—particularly those characterized by male sex or obesity—may inform targeted risk stratification approaches. However, prospective studies are required to determine whether interventions targeting arterial stiffness can delay CKD onset. Nevertheless, in daily clinical practice no calculation—including ePWV—can currently replace the direct assessment of serum creatinine and urine albumin-to-creatinine ratio for individuals identified as at risk of CKD on the basis of traditional risk factors.

In this study, LightGBM, XGBoost, and AdaBoost all demonstrated consistent and robust performance across the training, validation, and external testing sets. Formal DeLong tests showed no statistically significant differences between LightGBM and XGBoost in either internal validation (*p* = 0.0655) or external validation (*p* = 0.0684). Similar findings have been reported in studies on delayed decision-making in acute ischemic stroke, further supporting the stability and generalizability of these boosting algorithms [37]. Moreover, SHAP analysis not only confirmed a dose–response relationship for ePWV as a key feature but also revealed complex nonlinear interactions between ePWV and other clinical variables—patterns that are often beyond the explanatory capacity of traditional linear models. More broadly, the integration of quantitative modeling with biologically informative biomarkers and interpretable computational approaches provides a useful framework for translating complex biological signals into clinically meaningful risk assessment [38,39,40]. Comparable methodologies have been successfully applied in the classification of diabetic kidney disease [41].

These results suggest that ePWV may serve as a potentially useful marker for CKD risk assessment, with broad applicability across diverse populations and health states. Compared with conventional indicators, ePWV offers practical advantages: it is non-invasive, easy to calculate, cost-effective, and more suitable for primary care settings and remote risk assessment. When integrated with artificial intelligence algorithms, its incorporation into electronic health records and mobile health platforms is feasible, potentially enabling early identification and personalized management of CKD risk. 

However, it is important to emphasize that no non-invasive calculated metric can replace direct measurement of serum creatinine and urinalysis for identifying individuals at risk of CKD in routine clinical practice. ePWV should be regarded as a complementary tool for risk stratification rather than a substitute for established laboratory-based diagnostic methods.

### Limitations

However, several limitations warrant consideration. First, both the NHANES and CHARLS databases are cross-sectional in design, with exposure and outcome assessed at the same point in time; neither temporal sequence nor causality can be established. We therefore describe our findings in terms of association rather than prediction, and confirmation of the predictive value of ePWV requires prospective cohort studies. Second, measurement inconsistencies in certain covariates across the two datasets may introduce residual confounding. Third, CKD was defined by the full KDIGO criteria (eGFR and/or ACR) in NHANES but by the eGFR criterion alone in CHARLS, because urinary albumin was not measured in CHARLS; consequently, CKD prevalence, all descriptive data, and all model-derived estimates differ between the two cohorts by definition, and CHARLS may have missed individuals with albuminuria and preserved eGFR. This difference should be considered when interpreting cross-cohort comparisons. 

Fourth, all renal function assessments were based on a single measurement, without confirmation of chronicity. This may lead to misclassification of transient renal function changes as CKD. We therefore use the term “eGFR-defined CKD” for CHARLS analyses where appropriate to reflect this limitation. Fifth, ePWV remains an estimated surrogate marker; its agreement with directly measured carotid-femoral PWV (cfPWV) requires further validation across different populations. Sixth, antihypertensive medication use may influence both blood pressure and ePWV and was not fully adjusted for due to data availability; future studies with detailed medication records are needed to clarify this relationship. Finally, the generalizability of these machine learning findings to other populations or clinical settings requires further external validation.

## 5. Conclusions

This study demonstrates a significant association between elevated estimated pulse wave velocity (ePWV) and higher odds of eGFR-defined CKD in both U.S. and Chinese populations. ePWV showed incremental discriminative value beyond age and blood pressure alone and ranked highest in model-derived feature importance among the machine learning features evaluated. These findings support ePWV as a potentially useful marker for CKD risk stratification; however, prospective studies are required to establish its utility for predicting incident CKD and guiding preventive interventions.

## Figures and Tables

**Table 1:** Baseline Characteristics of the NHANES Participants.

Characteristic	Overall *N* = 100,907,775	CKD		
		No *N* = 79,303,257	Yes *N* = 21,604,517	*p*-Value
Age	60.30 (10.53)	58.34 (9.52)	67.48 (10.93)	<0.001
PIR	3.19 (1.56)	3.32 (1.55)	2.74 (1.53)	<0.001
BMI	29.30 (6.40)	28.25 [24.80, 32.29]	29.05 [25.40, 33.54]	NA
ALT	24.80 (18.80)	22.00 [17.00, 28.00]	20.00 [15.00, 26.00]	NA
AST	25.74 (14.88)	23.00 [20.00, 28.00]	24.00 [20.00, 28.00]	NA
UA	5.52 (1.40)	5.37 (1.30)	6.08 (1.58)	<0.001
TC	199.35 (42.73)	201.14 (41.59)	192.78 (46.08)	<0.001
TG	132.18 (70.43)	132.28 [107.00, 132.28]	132.28 [122.00, 132.28]	NA
LDL	116.19 (25.09)	117.36 (24.66)	111.91 (26.19)	<0.001
SBP	127.15 (18.07)	125.18 (16.43)	134.39 (21.62)	<0.001
MBP	93.47 (11.83)	93.15 (11.03)	94.66 (14.34)	<0.001
ePWV	9.44 (1.88)	9.09 (1.68)	10.73 (1.99)	<0.001
DBP	71.02 (12.47)	71.80 (11.57)	68.17 (14.98)	<0.001
eGFR	82.73 (18.88)	87.54 (14.02)	65.07 (23.37)	<0.001
Gender, *n* (*p*%)				0.004
Male	48,713,957 (48.28%)	38,937,261.5 (49.1%)	9,776,695.1 (45.3%)	
Female	52,193,818 (51.72%)	40,365,995.6 (50.9%)	11,827,822.4 (54.7%)	
Race, *n* (*p*%)				<0.001
Mexican American	5,609,215 (5.56%)	4,473,487.3 (5.6%)	1,135,727.4 (5.3%)	
Non-Hispanic Black	4,251,644(4.21%)	3,435,663.0 (4.3%)	815,980.7 (3.8%)	
Non-Hispanic White	75,005,723 (74.33%)	59,159,072.2 (74.6%)	15,846,650.5 (73.3%)	
Other Hispanic	9,465,340 (9.38%)	6,953,472.9 (8.8%)	2,511,867.3 (11.6%)	
Other race	6,575,853 (6.52%)	5,281,561.7 (6.7%)	1,294,291.7 (6.0%)	
Education, *n* (*p*%)				<0.001
Above high school	10,151,401 (10.06%)	7,305,088.4 (9.2%)	2,846,312.6 (13.2%)	
High school	24,069,712 (23.85%)	18,410,463.4 (23.2%)	5,659,248.3 (26.2%)	
Under high school	66,686,662 (66.09%)	53,587,705.3 (67.6%)	13,098,956.6 (60.6%)	
Marital, *n* (*p*%)				<0.001
Living with a partner	64,039,452 (63.46%)	52,397,569.6 (66.1%)	11,641,882.3 (53.9%)	
Living alone	36,868,323 (36.54%)	26,905,687.5 (33.9%)	9,962,635.2 (46.1%)	
PIR_Group, *n* (*p*%)				<0.001
<1.30	9,957,381 (9.87%)	7,071,495.1 (8.9%)	2,885,885.8 (13.4%)	
1.30~3.49	51,654,267 (51.19%)	38,833,148.0 (49.0%)	12,821,118.9 (59.3%)	
≥3.50	39,296,127 (38.94%)	33,398,613.9 (42.1%)	5,897,512.7 (27.3%)	
BMI_Group, *n* (*p*%)				<0.001
Normal	25,451,380 (25.22%)	20,622,875.0 (26.0%)	4,828,505.4 (22.3%)	
Overweight	36,385,252 (36.06%)	28,943,652.1 (36.5%)	7,441,599.9 (34.4%)	
Obese	39,071,142 (38.72%)	29,736,730.0 (37.5%)	9,334,411.7 (43.2%)	
Drink				<0.001
No	26,675,556 (26.44%)	9,334,411.7 (43.2%)	7,033,078.8 (32.6%)	
Yes	74,232,219 (73.56%)	59,660,780.1 (75.2%)	14,571,438.7 (67.4%)	
Hypertension				<0.001
No	54,506,764 (54.02%)	47,513,837.8 (59.9%)	6,992,926.2 (32.4%)	
Yes	46,401,011 (45.98%)	31,789,419.3 (40.1%)	14,611,591.3 (67.6%)	
Dyslipidemia				<0.001
No	50,670,504 (50.21%)	41,071,601.4 (51.8%)	9,598,903.0 (44.4%)	
Yes	50,237,270 (49.79%)	38,231,655.7 (48.2%)	12,005,614.5 (55.6%)	
Diabetes				<0.001
No	85,038,766 (84.27%)	69,957,357.1 (88.2%)	15,081,409.4 (69.8%)	
Yes	15,869,008 (15.73%)	9,345,900.0 (11.8%)	6,523,108.1 (30.2%)	
PA				<0.001
No	57,649,602 (57.13%)	43,320,678.6 (54.6%)	14,328,923.7 (66.3%)	
Yes	43,258,172 (42.87%)	35,982,578.5 (45.4%)	7,275,593.8 (33.7%)	
Smoke				0.02
No	51,477,025 (51.01%)	41,121,288.6 (51.9%)	10,355,736.8 (47.9%)	
Yes	49,430,749 (48.99%)	38,181,968.5 (48.1%)	11,248,780.7 (52.1%)	
ePWV, *n*				<0.001
Q1	31,500,824 (31.22%)	28,788,219.0 (36.3%)	2,712,604.8 (12.6%)	
Q2	27,470,665 (27.22%)	23,618,252.3 (29.8%)	3,852,412.7 (17.8%)	
Q3	23,112,660 (22.90%)	17,268,056.5 (21.8%)	5,844,603.4 (27.1%)	
Q4	18,823,626 (18.65%)	9,628,729.2 (12.1%)	9,194,896.6 (42.6%)	

**Table 2:** Baseline Characteristics of the CHARLS Participants.

		CKD		
Variable	Overall	No	Yes	*p*-Value
	*N* = 21,853	*N* = 21,107	*N* = 746	
Age, mean (sd)	59.98 (9.37)	59.66 (9.21)	68.93 (9.44)	<0.001
ePWV, mean (sd)	9.63 (1.88)	9.57 (1.85)	11.27 (1.96)	<0.001
eGFR, mean (sd)	94.63 (15.29)	96.34 (12.29)	46.11 (11.68)	<0.001
Gender, *n* (*p*%)				0.197
Male	10,244.00 (46.88%)	9877.00 (46.79%)	367.00 (49.20%)	
Female	11,609.00 (53.12%)	11,230.00 (53.21%)	379.00 (50.80%)	
Marital, *n* (*p*%)				<0.001
Living with a partner Living alone	18,203.00 (83.30%)	17,646.00 (83.60%)	557.00 (74.66%)	
	3650.00 (16.70%)	3461.00 (16.40%)	189.00 (25.34%)	
Education, *n* (*p*%)				<0.001
Illiterate	5961.00 (27.28%)	5699.00 (27.00%)	262.00 (35.12%)	
Junior high school or below	13,657.00 (62.49%)	13,223.00 (62.65%)	434.00 (58.18%)	
High school or above	2235.00 (10.23%)	2185.00 (10.35%)	50.00 (6.70%)	
Residence, *n* (*p*%)				<0.001
Rural	13,841.00 (63.34%)	13,426.00 (63.61%)	415.00 (55.63%)	
Urban	8012.00 (36.66%)	7681.00 (36.39%)	331.00 (44.37%)	
Hypertension, *n* (*p*%)				<0.001
No	15,237.00 (69.72%)	14,864.00 (70.42%)	373.00 (50.0%)	
Yes	6616.00 (30.28%)	6243.00(29.58%)	373.00 (50.0%)	
Dyslipidemia, *n* (*p*%)				<0.001
No	18,820.00 (86.12%)	18,220.00 (86.32%)	600.00 (80.43%)	
Yes	3033.00 (13.88%)	2887.00 (13.68%)	146.00 (19.57%)	
Diabetes, *n* (*p*%)				<0.001
No	20,055.00 (91.77%)	19,422.00 (92.02%)	633.00 (84.85%)	
Yes	1798.00 (8.23%)	1685.00 (7.98%)	113.00 (15.15%)	
ePWV, *n* (*p*%)				<0.001
Q1	5463.00 (25.00%)	5406.00 (25.61%)	57.00 (7.64%)	
Q2	5463.00 (25.00%)	5372.00 (25.45%)	91.00(12.20%)	
Q3	5463.00 (25.00%)	5314.00 (25.18%)	149.00 (19.97%)	
Q4	5464.00 (25.00%)	5015.00 (23.76%)	449.00 (60.19%)	
Smoke, *n* (*p*%)				0.350
No	13,074.00 (59.83%)	12,640.00 (59.89%)	434.00 (58.18%)	
Yes	8779.00 (40.17%)	8467.00 (40.11%)	312.00 (41.82%)	
Drink, *n* (*p*%)				0.497
No	12,214.00 (55.89%)	11,788.00 (55.85%)	426.00 (57.10%)	
Yes	9639.00 (44.11%)	9319.00 (44.15%)	320.00 (42.90%)	
BMI, *n* (*p*%)				0.101
Normal	12,351.00 (56.52%)	11,919.00 (56.47%)	432.00 (57.91%)	
Overweight	7290.00 (33.36%)	7064.00 (33.47%)	226.00 (30.29%)	
Obese	2212.00 (10.12%)	2124.00 (10.06%)	88.00 (11.80%)	

The ePWV quartile cutoffs were, in NHANES, Q1 < 8.265, Q2 < 9.564, Q3 < 11.233, and Q4 ≥ 11.233 m/s; in CHARLS, Q1 < 8.192, Q2 < 9.411, Q3 < 10.875, and Q4 ≥ 10.875 m/s.

**Table 3:** Association between ePWV and CKD in the NHANES and CHARLS population.

Cohort	Model	Variable	OR	95% CI	*p*
NHANES	Model 1	ePWV (per 1 m/s)	1.599	(1.553, 1.647)	<0.001
		Q2	1.73	(1.46, 2.06)	<0.001
		Q3	3.59	(3.03, 4.26)	<0.001
		Q4	10.13	(8.56, 12.00)	<0.001
		*p for trend*			<0.001
	Model 2	ePWV (per 1 m/s)	1.565	(1.519, 1.613)	<0.001
		Q2	1.74	(1.46, 2.06)	<0.001
		Q3	3.53	(2.98, 4.19)	<0.001
		Q4	9.22	(7.79, 10.92)	<0.001
		*p for trend*			<0.001
	Model 3	ePWV (per 1 m/s)	1.505	(1.459, 1.552)	<0.001
		Q2	1.45	(1.22, 1.73)	<0.001
		Q3	2.71	(2.28, 3.23)	<0.001
		Q4	7.02	(5.93, 8.31)	<0.001
		*p for trend*			<0.001
CHARLS	Model 1	ePWV (per 1 m/s)	1.556	(1.495, 1.618)	<0.001
		Q2	1.61	(1.15, 2.24)	0.005
		Q3	2.66	(1.95, 3.63)	<0.001
		Q4	8.49	(6.39, 11.29)	<0.001
		*p for trend*			<0.001
	Model 2	ePWV (per 1 m/s)	1.543	(1.478, 1.611)	<0.001
		Q2	1.57	(1.13, 2.19)	0.007
		Q3	2.55	(1.87, 3.48)	<0.001
		Q4	7.77	(5.83, 10.36)	<0.001
		*p for trend*			<0.001
	Model 3	ePWV (per 1 m/s)	1.434	(1.368, 1.503)	<0.001
		Q2	1.37	(0.97, 1.92)	0.072
		Q3	1.86	(1.35, 2.57)	<0.001
		Q4	4.99	(3.71, 6.71)	<0.001
		*p for trend*			<0.001

Model 1: Unadjusted. Model 2: Adjusted for sex, residence, marital status, and education level. Model 3: Additionally adjusted for uric acid, triglycerides, hemoglobin, total cholesterol, BMI, LDL-C, hypertension, dyslipidemia, diabetes, alcohol consumption, smoking status, and MET. SBP, DBP, and MBP were excluded from Model 3, as they are already incorporated into the ePWV calculation. Abbreviations: OR, odds ratio; CI, confidence interval.

**Table 4:** Subgroup analyses for the association between ePWV and CKD, with interaction *p* values.

Cohort	Subgroup	Level	*n*	CKD_*n*	OR (95% CI)	*p*	*p*_Interaction
NHANES	Gender	Male	9008	2417	1.579 (1.507, 1.655)	<0.001	0.012
		Female	8882	2381	1.441 (1.381, 1.504)	<0.001	
	BMI	Normal	4372	1070	1.435 (1.369, 1.503)	<0.001	0.025
		Overweight	6518	1694	1.558 (1.478, 1.642)	<0.001	
		Obese	7000	2034	1.523 (1.460, 1.588)	<0.001	
	Smoke	No	9025	2293	1.489 (1.427, 1.555)	<0.001	0.437
		Yes	8865	2505	1.525 (1.462, 1.591)	<0.001	
	Drink	No	5523	1667	1.489 (1.415, 1.568)	<0.001	0.947
		Yes	12,367	3131	1.512 (1.456, 1.569)	<0.001	
	Hypertension	No	8768	1443	1.489 (1.416, 1.565)	<0.001	0.676
		Yes	9122	3355	1.517 (1.461, 1.576)	<0.001	
	Dyslipidemia	No	8833	2120	1.505 (1.443, 1.569)	<0.001	0.862
		Yes	9057	2678	1.506 (1.448, 1.566)	<0.001	
	Diabetes	No	14,191	3098	1.523 (1.473, 1.574)	<0.001	0.006
		Yes	3699	1700	1.446 (1.366, 1.532)	<0.001	
CHARLS	Gender	Male	10,244	367	1.553 (1.453, 1.659)	<0.001	<0.001
		Female	11,609	379	1.318 (1.233, 1.409)	<0.001	
	BMI	Normal	12,351	432	1.475 (1.387, 1.570)	<0.001	0.108
		Overweight	7290	226	1.306 (1.202, 1.420)	<0.001	
		Obese	2212	88	1.634 (1.411, 1.892)	<0.001	
	Smoke	No	13,074	432	1.374 (1.292, 1.461)	<0.001	0.068
		Yes	8779	314	1.521 (1.411, 1.640)	<0.001	
	Drink	No	12,214	429	1.408 (1.324, 1.498)	<0.001	0.544
		Yes	9639	317	1.469 (1.364, 1.582)	<0.001	
	Hypertension	No	15,238	370	1.444 (1.357, 1.535)	<0.001	0.751
		Yes	6615	376	1.425 (1.324, 1.533)	<0.001	
	Dyslipidemia	No	18,820	598	1.445 (1.371, 1.523)	<0.001	0.428
		Yes	3033	148	1.393 (1.256, 1.544)	<0.001	
	Diabetes	No	20,054	631	1.448 (1.377, 1.522)	<0.001	0.479
		Yes	1799	115	1.350 (1.182, 1.541)	<0.001	

Notes: ORs are from Model 3 (fully adjusted, excluding SBP/DBP/MBP).

**Table 5:** Restricted cubic spline analysis for the association between ePWV and CKD.

Cohort	Knots	Knot Locations	Reference ePWV	*p* for Nonlinearity	*p* for Overall
NHANES	4	7.017, 8.783, 10.461, 13.265	9.564	<0.001	<0.001
CHARLS	4	6.913, 8.683, 10.216, 13.095	9.411	0.071	<0.001

Notes: Models were adjusted for covariates in Model 3 (excluding SBP/DBP/MBP, as these are already incorporated into the ePWV calculation). Knots were placed at the 5th, 35th, 65th, and 95th percentiles of the ePWV distribution in each cohort. The reference value was set at the median ePWV in each cohort. *p* for nonlinearity tests the deviation from a linear relationship; *p* for overall tests the overall association between ePWV and CKD.

**Table 6:** Age-adjusted analysis of the association between ePWV and CKD.

Cohort	Model	OR	95% CI	*p*
NHANES	Model 3 (no BP) + Age	1.294	(1.244, 1.347)	<0.001
CHARLS	Model 3 (no BP) + Age	0.997	(0.921, 1.080)	0.947

Notes: Model 3 was additionally adjusted for age. In CHARLS, an OR of 0.997 indicates that after accounting for age, the residual association between ePWV and CKD was largely attenuated, reflecting the contribution of age to the ePWV calculation. Abbreviations: OR, odds ratio; CI, confidence interval.

**Table 7:** Comparison of AUROCs for models containing age, blood pressure, ePWV, and their combination.

Cohort	Model	AUROC (95% CI)	DeLong *p* (ePWV vs. Age)
NHANES	Age only	0.726 (0.717, 0.734)	0.29
NHANES	Blood pressure only	0.528 (0.518, 0.538)	—
NHANES	ePWV only	0.728 (0.720, 0.737)	0.29
NHANES	Combined (age + BP + ePWV)	0.735 (0.727, 0.744)	—
CHARLS	Age only	0.759 (0.741, 0.777)	0.000166
CHARLS	Blood pressure only	0.542 (0.521, 0.564)	—
CHARLS	ePWV only	0.737 (0.718, 0.756)	0.000166
CHARLS	Combined (age + BP + ePWV)	0.761 (0.743, 0.779)	—

Notes: DeLong test compared ePWV-only vs. age-only models. In CHARLS, the age-only model showed a statistically significantly higher AUROC than the ePWV-only model (0.759 vs. 0.737; *p* = 0.000166); in NHANES, the difference was not significant (*p* = 0.29). Abbreviations: AUROC, area under the receiver operating characteristic curve; BP, blood pressure; CI, confidence interval.

**Table 8:** Net reclassification improvement (NRI) and integrated discrimination improvement (IDI) for adding ePWV to age-based models.

Cohort	Comparison	NRI (95% CI)	NRI *p*	IDI (95% CI)	IDI *p*
NHANES	Age → Combined	0.1856 (0.1517, 0.2204)	<0.001	0.0145 (0.0128, 0.0164)	<0.001
NHANES	ePWV → Combined	0.1546 (0.1228, 0.1898)	<0.001	0.0132 (0.0113, 0.0152)	<0.001
CHARLS	Age → Combined	0.0094 (−0.0633, 0.0861)	0.800	0.0007 (0.0003, 0.0013)	0.004
CHARLS	ePWV → Combined	0.3984 (0.3300, 0.4646)	<0.001	0.0097 (0.0074, 0.0120)	<0.001

Notes: ‘Combined’ refers to the model containing age, blood pressure, and ePWV together. NRI > 0 indicates net improvement in reclassification; IDI > 0 indicates improved overall discrimination. In CHARLS, although the NRI for adding ePWV to age was not statistically significant (*p* = 0.800), the significant IDI (*p* = 0.004) suggests a modest incremental value. Abbreviations: NRI, net reclassification improvement; IDI, integrated discrimination improvement; CI, confidence interval.

**Table 9:** Optimal cut-off values of ePWV for discriminating CKD (Youden index).

Cohort	AUROC (95% CI)	Best Cut-Off (m/s)	Sensitivity	Specificity
NHANES	0.728 (0.720, 0.737)	10.15	0.658	0.695
CHARLS	0.737 (0.718, 0.756)	10.568	0.658	0.718

Notes: Optimal cut-off values were determined using the Youden index (maximizing sensitivity + specificity − 1). The best cut-off for NHANES was 10.15 m/s; and for CHARLS was 10.568 m/s.

**Table 10:** Performance of machine learning models in the NHANES validation set.

Model	Accuracy	AUROC	F1	MCC	Sensitivity	Specificity	Brier
RF	0.789 (0.777, 0.800)	0.808 (0.794, 0.821)	0.530 (0.501, 0.558)	—	0.441 (0.412, 0.467)	0.918 (0.910, 0.926)	0.147 (0.141, 0.153)
XGBoost	0.754 (0.743, 0.767)	0.801 (0.787, 0.815)	0.593 (0.573, 0.615)	—	0.662 (0.636, 0.684)	0.788 (0.776, 0.802)	0.168 (0.162, 0.173)
LightGBM	0.748 (0.737, 0.760)	0.804 (0.791, 0.818)	0.596 (0.577, 0.616)	—	0.685 (0.660, 0.709)	0.771 (0.758, 0.785)	0.171 (0.165, 0.176)
AdaBoost	0.793 (0.781, 0.804)	0.812 (0.798, 0.825)	0.544 (0.517, 0.570)	—	0.456 (0.426, 0.483)	0.918 (0.910, 0.926)	0.145 (0.139, 0.151)
Naive Bayes	0.778 (0.767, 0.789)	0.780 (0.766, 0.793)	0.479 (0.452, 0.503)	—	0.376 (0.349, 0.402)	0.928 (0.919, 0.936)	0.172 (0.164, 0.181)
Decision Tree	0.783 (0.772, 0.794)	0.771 (0.756, 0.785)	0.534 (0.508, 0.557)	—	0.458 (0.432, 0.484)	0.904 (0.894, 0.913)	0.157 (0.150, 0.164)

Notes: Values are presented as point estimates with 95% confidence intervals in parentheses. MCC values were not available due to software output limitations. The NHANES dataset was randomly split into training (70%) and validation (30%) sets. Abbreviations: AUROC, area under the receiver operating characteristic curve; MCC, Matthews correlation coefficient; RF, Random Forest.

**Table 11:** Performance of machine learning models in the CHARLS external validation set.

Model	Accuracy	AUROC	F1	MCC	Sensitivity	Specificity	Brier
RF	0.931 (0.928, 0.935)	0.776 (0.758, 0.794)	0.246 (0.220, 0.269)	—	0.328 (0.294, 0.359)	0.952 (0.949, 0.955)	0.070 (0.069, 0.072)
XGBoost	0.862 (0.858, 0.866)	0.788 (0.771, 0.805)	0.208 (0.191, 0.224)	—	0.529 (0.494, 0.564)	0.874 (0.870, 0.878)	0.109 (0.107, 0.111)
LightGBM	0.851 (0.846, 0.856)	0.793 (0.775, 0.809)	0.208 (0.191, 0.223)	—	0.571 (0.536, 0.607)	0.861 (0.856, 0.866)	0.117 (0.115, 0.119)
AdaBoost	0.940 (0.937, 0.943)	0.800 (0.784, 0.817)	0.279 (0.248, 0.305)	—	0.338 (0.302, 0.372)	0.962 (0.959, 0.964)	0.057 (0.055, 0.058)
Naive Bayes	0.945 (0.942, 0.948)	0.757 (0.740, 0.775)	0.178 (0.153, 0.207)	—	0.176 (0.148, 0.207)	0.972 (0.970, 0.974)	0.045 (0.043, 0.047)
Decision Tree	0.920 (0.916, 0.924)	0.757 (0.740, 0.776)	0.248 (0.224, 0.269)	—	0.385 (0.349, 0.419)	0.939 (0.936, 0.943)	0.072 (0.070, 0.074)

Notes: Values are presented as point estimates with 95% confidence intervals in parentheses. MCC values were not available due to software output limitations. The CHARLS dataset (*n* = 21,853) was used as an external validation set without any model retraining. Abbreviations: AUROC, area under the receiver operating characteristic curve; MCC, Matthews correlation coefficient; RF, Random Forest.

**Table 12:** Five-fold cross-validation performance of LightGBM in the NHANES full sample.

Method	AUROC Mean	95% CI	Fold 1	Fold 2	Fold 3	Fold 4	Fold 5
LightGBM 5-fold CV	0.7985	(0.7911, 0.8045)	0.7962	0.8043	0.8062	0.8017	0.7844

Notes: Five-fold cross-validation was performed on the full NHANES sample (*n* = 17,890) to assess model robustness. The narrow confidence interval and consistent performance across folds indicate good stability. Abbreviations: CV, cross-validation.

**Table 13:** DeLong test comparing LightGBM and XGBoost.

Comparison	Internal Validation *p*	External Validation *p*
LightGBM vs. XGBoost	0.0655	0.0684

Notes: Both *p* values are >0.05, indicating no statistically significant difference in AUROC between LightGBM and XGBoost in either internal or external validation. This supports a neutral interpretation that LightGBM performed comparably to XGBoost rather than demonstrating superiority.

**Table 14:** SHAP feature importance for the CKD classification model.

Rank	Feature	Mean Absolute SHAP	Proportion (%)
1	ePWV	0.489	16.13
2	UA	0.438	14.45
3	Diabetes	0.346	11.42
4	Age	0.294	9.71
5	Hypertension	0.234	7.73
6	SBP	0.147	4.86
7	DBP	0.143	4.71
8	TC	0.136	4.49
9	Marital	0.108	3.56
10	Gender	0.105	3.45
11	LDL	0.103	3.39
12	TG	0.098	3.24
13	MAP	0.097	3.19
14	Education	0.054	1.76
15	BMI_grp2	0.045	1.47
16	BMI_grp3	0.040	1.33
17	Dyslipidemia	0.040	1.32
18	Drinking	0.035	1.14
19	Smoking	0.031	1.01
20	Education2	0.025	0.82
21	Gender2	0.024	0.80

Notes: SHAP values represent the average absolute contribution of each feature to model predictions across all samples. Among all 21 included features, ePWV ranked highest in model-derived feature importance (16.13%), surpassing traditional risk factors such as uric acid (14.45%), diabetes (11.42%), age (9.71%), and hypertension (7.73%). Abbreviation: SHAP, SHapley Additive exPlanations.

## Data Availability

The authors accessed the NHANES (https://wwwn.cdc.gov/nchs/nhanes/, accessed on 3 August 2025) and CHARLS (http://charls.pku.edu.cn/, accessed on 3 August 2025) data for this study on 3 August 2025, from their respective official websites.

## Supplementary Materials

The following supporting information can be downloaded at: https://www.mdpi.com/article/10.3390/healthcare14183125/s1, Supplementary Figure S1: ROC curves of ePWV for discriminating CKD in NHANES and CHARLS; Supplementary Figure S2: Precision-recall curves for machine learning models; Supplementary Figure S3: Calibration curves for machine learning models; Supplementary Figure S4: Decision curve analysis for machine learning models; Supplementary Figure S5: Residual Q–Q plots for machine learning models.

## Abbreviations

The following abbreviations are used in this manuscript:

CKD	chronic kidney disease
ePWV	Estimated pulse wave velocity
NHANES	National Health and Nutrition Examination Survey
CHARLS	China Health and Retirement Longitudinal Study
SHAP	SHapley Additive exPlanations
ESRD	end-stage renal disease
GBD	global burden of disease
